# Supplementary material for: Differences in three-dimensional kinetic determinants of jump height between single- and double-leg countermovement jumps
Source: Biol Open. 2026 Apr 30;15(4):bio062434. doi: 10.1242/bio.062434 (PMC13225229; doi:10.1242/bio.062434)
Supplement: Supplementary information [file biolopen-15-062434-s1.pdf]

Statistical results of spm-1d linear regression analyses

The results of the spm1d linear regression analyses between the kinetic variables and jump height in the single-leg and double-leg CMJs are presented in Figures S1–S4. Figure S1 and S2 correspond to the statistical results for Figure 6 in the main text, and Figure S3 and S4 correspond to the statistical results for Figure 7. In all figures, the gray-shaded areas indicate the time ranges where the t-curves exceeded the threshold (red dotted line), representing significant correlations between the kinetic variables and jump height. Figure S1 and Figure S2 show the results for the lower-limb joints in the single-leg and double-leg CMJs, respectively. Figure S3 and Figure S4 show the results for the lumbosacral joint in the single-leg and double-leg CMJs, respectively.

Biology Open • Supplementary information

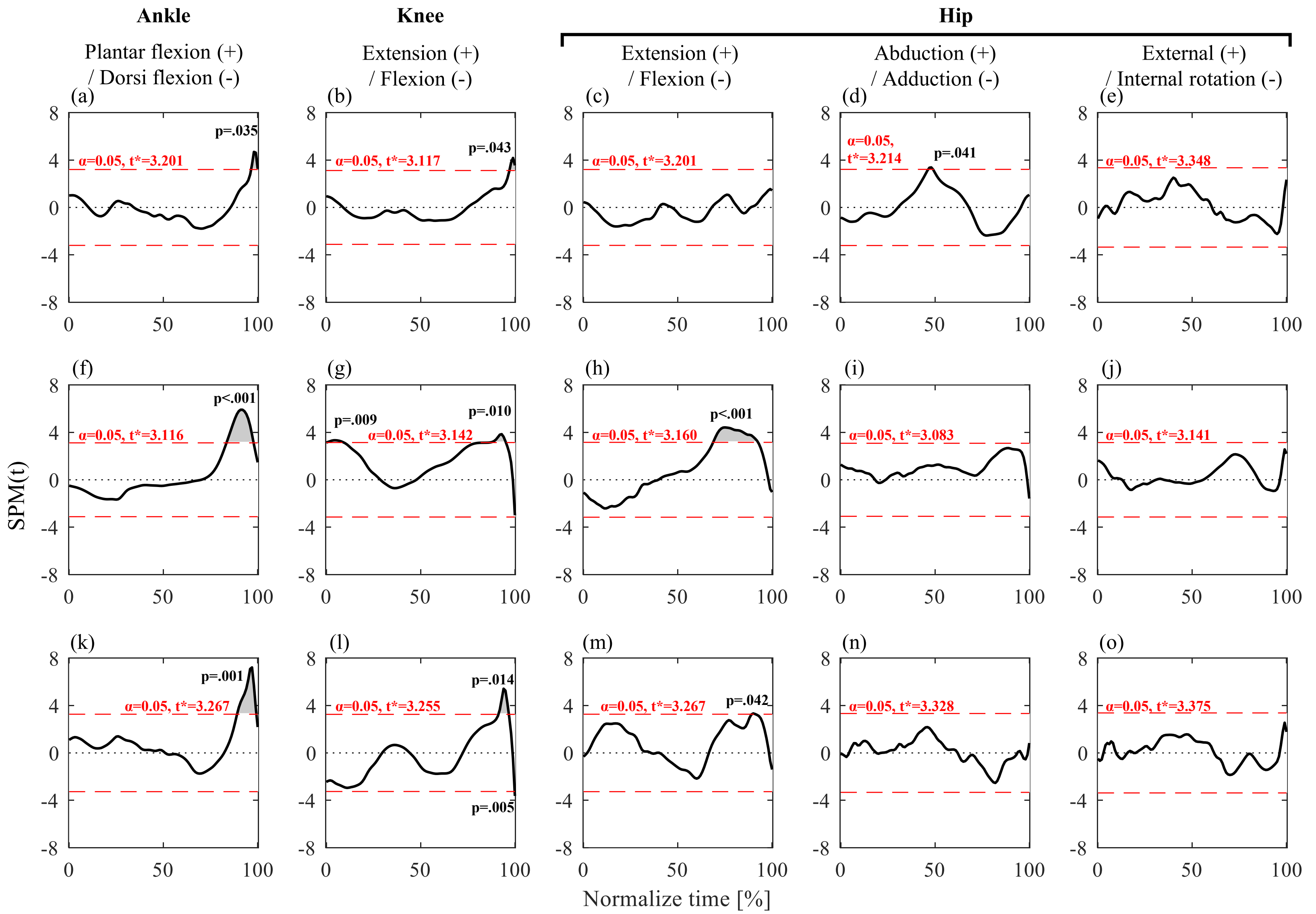

Fig. S1. The statistical results for joint angular velocities (a-e), torques (f-j), powers (k-o) of the ankle (a, f, k), knee (b, g, l), and hip (c-e, h-j, m-o) joints during the single-leg CMJ.

Biology Open • Supplementary information

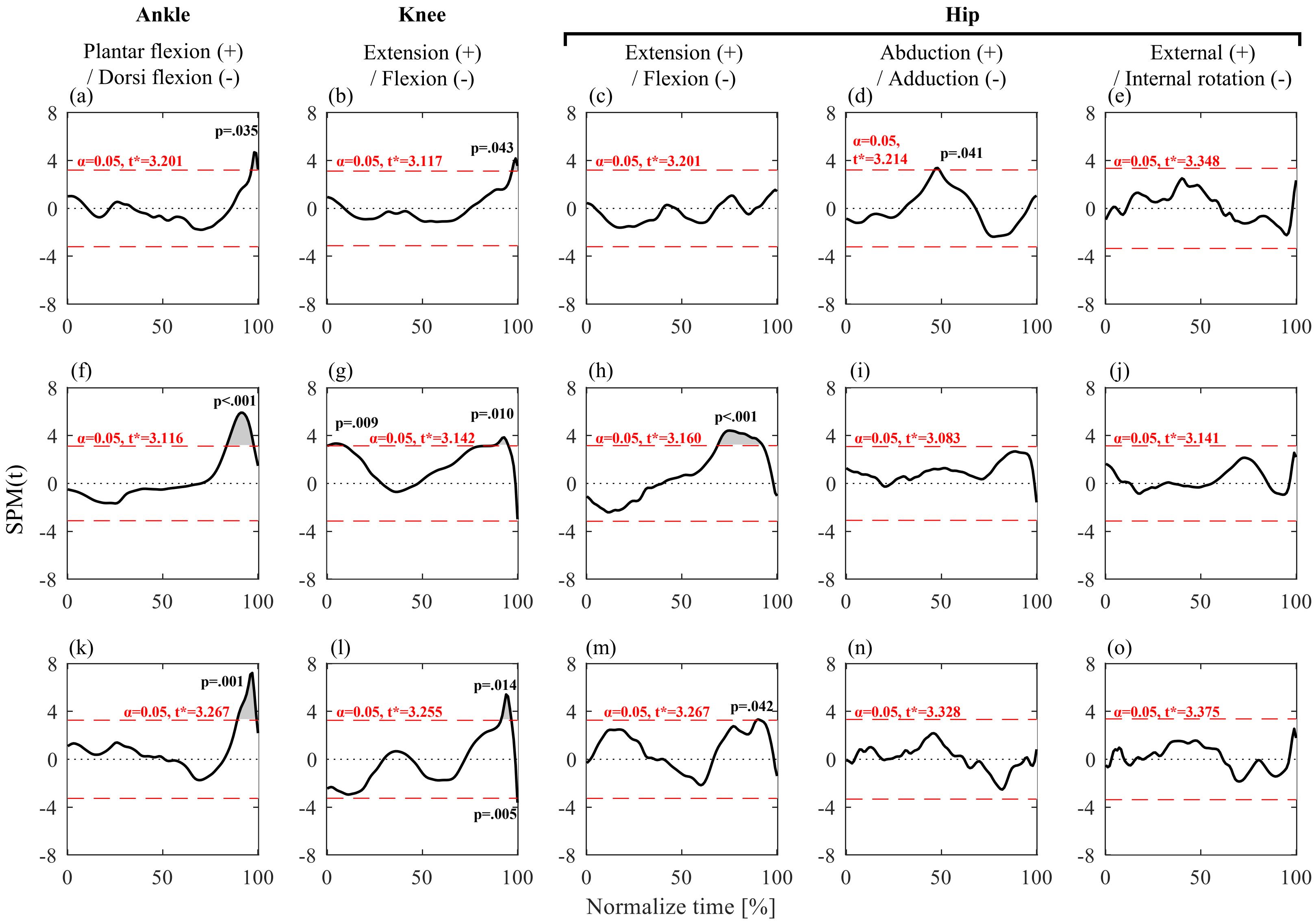

Fig. S2. The statistical results for joint angular velocities (a-e), torques (f-j), powers (k-o) of the ankle (a, f, k), knee (b, g, l), and hip (c-e, h-j, m-o) joints during the double-leg CMJ.

Biology Open • Supplementary information

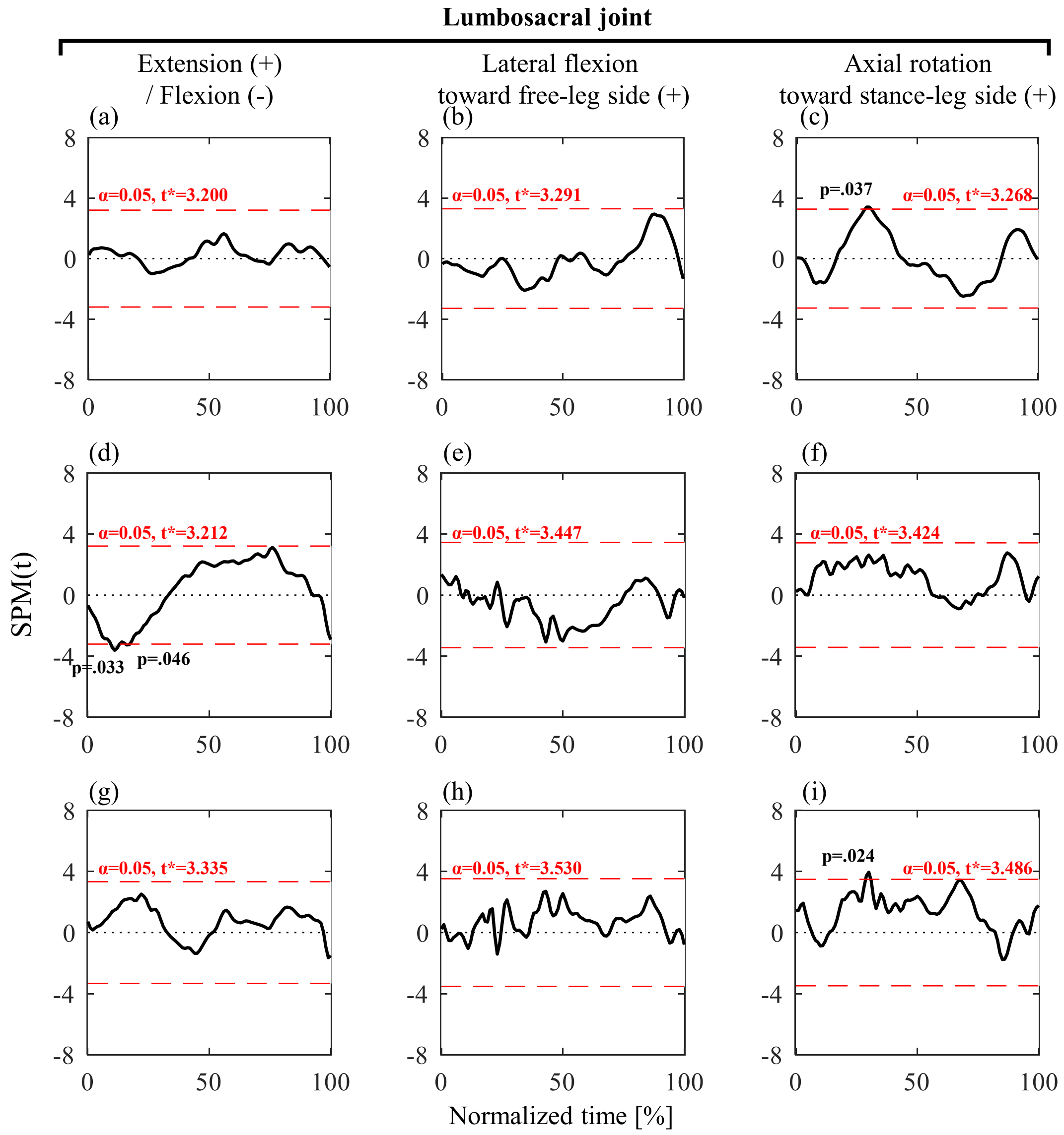

Fig. S3. The statistical results for joint angular velocities (a-c), torques (d-f), powers (g-i) of the lumbosacral joints during the single-leg CMJ.

Biology Open • Supplementary information

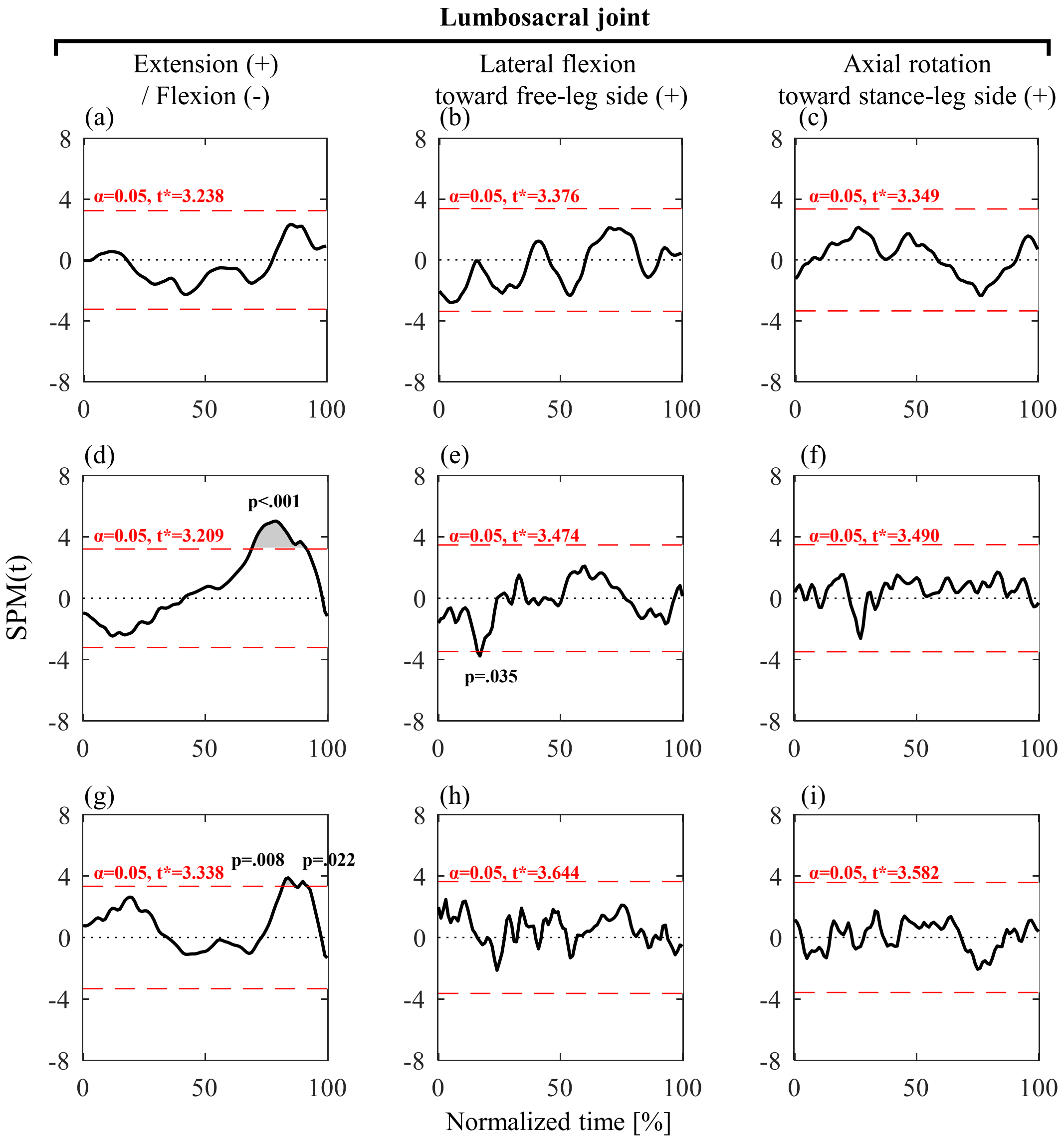

Fig. S4. The statistical results for joint angular velocities (a-c), torques (d-f), powers (g-i) of the lumbosacral joints during the double-leg CMJ.

Biology Open • Supplementary information

Results for the single-leg CMJ on the inferior leg side

The results of the spm1d linear regression analyses between the kinetic variables and jump height in the single-leg CMJ on the inferior leg side (Figure S5–S8). Additionally, the correlation coefficients (r) and p-values between the jump height and peak joint torque and power of lower-limb and lumbosacral joints during the single-leg CMJ are presented in Table S1. In all figures, the gray-shaded areas indicate the time ranges where the t-curves exceeded the threshold (red dotted line), representing significant correlations between the kinetic variables and jump height.

Table S1. Correlation coefficients (r) and p-values between the jump height and peak joint torque and power of lower-limb and lumbosacral joints during the single-leg CMJ on the inferior leg side.

		Single-leg CMJ	
		r	p-value
peak torque (unit, Nm/kg)	Ankle plantarflexion (+)	**0.731**	**<0.001**
	Knee extension (+)	**0.628**	**<0.001**
	Hip extension (+)	**0.462**	**0.001**
	Hip abduction (+)	**0.640**	**<0.001**
	Hip external rotation (+)	—	—
	Hip internal rotation (-)	**-0.440**	**0.002**
	Lumbosacral extension (+)	**0.409**	**0.004**
	Lumbosacral free-side lateral flexion (+)	**0.317**	**0.028**
	Lumbosacral free-side rotation (+)	**0.371**	**0.009**
peak positive power (unit, W/kg)	Ankle plantar-dorsiflexion axis	**0.769**	**<0.001**
	Knee extension-flexion axis	**0.677**	**<0.001**
	Hip extension-flexion axis	**0.472**	**0.001**
	Hip abduction-adduction axis	**0.576**	**<0.001**
	Hip external-internal rotation axis	**0.565**	**<0.001**
	Lumbosacral extension-flexion axis	**0.404**	**0.004**
	Lumbosacral lateral-flexion axis	**0.391**	**0.006**
	Lumbosacral rotation axis	0.141	0.338

The bold font indicates a statistically significant correlation ($p < 0.05$). The correlation results of the hip external rotation torque for single-leg CMJ are not shown because these torques were not produced (see Figure S5).

Biology Open • Supplementary information

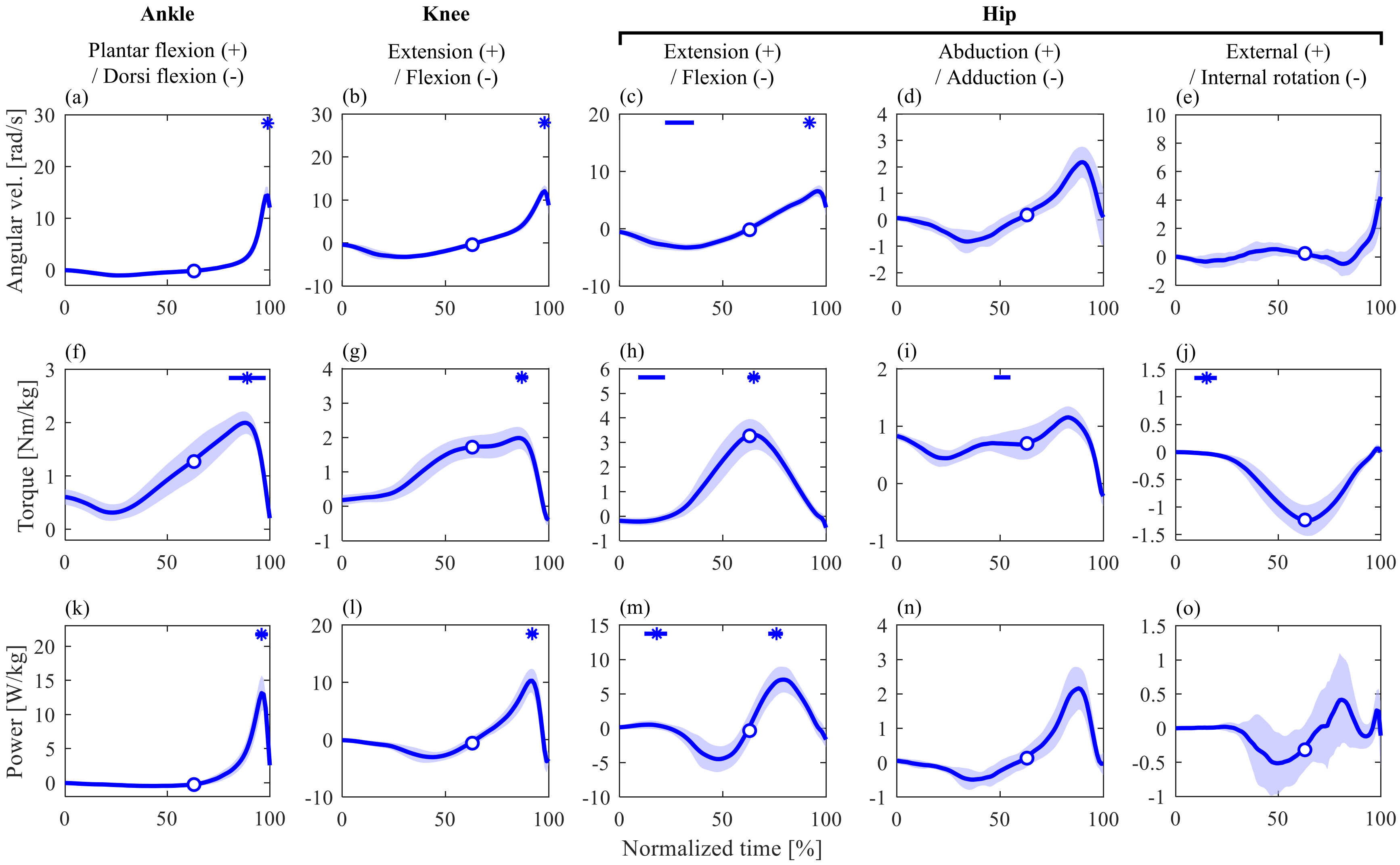

Fig. S5. Joint angular velocities, torques, and powers of the ankle (a, f, and k), knee (b, g, and l), and hip (c–e, h–j, and m–o) joints during the single-leg CMJ on the inferior leg side. The circle markers indicate when the CoM velocity was zero, representing the transition from the unweighting to the propulsive phase. The asterisks (*) and horizontal bars at the top of each graph indicate the time points showing significant correlations with jump height. The asterisks and bars represent positive correlations, whereas the bars alone represent negative correlations.

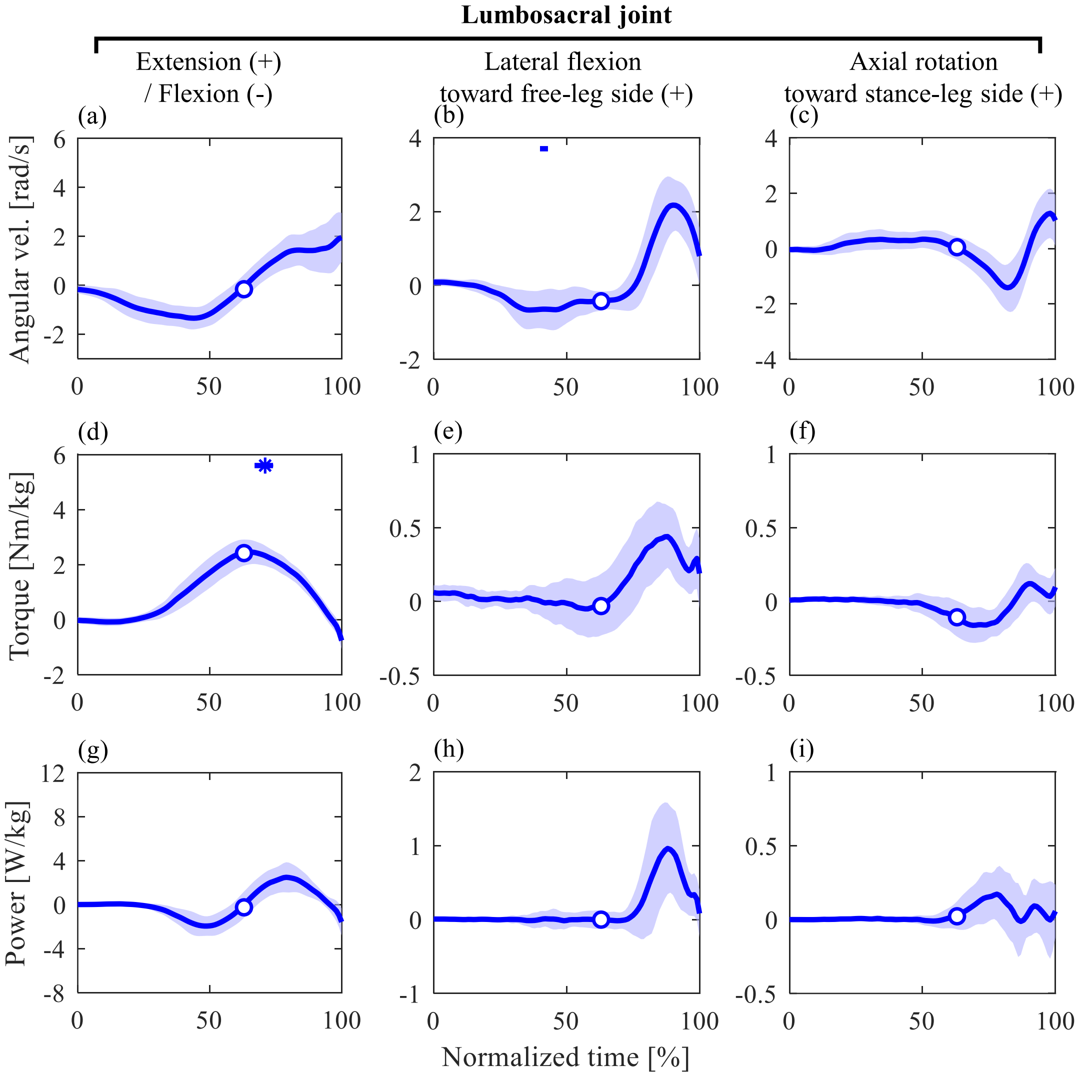

Fig. S6. Joint angular velocities, torques, and powers of the lumbosacral joint during the single-leg CMJ on the inferior leg side. The circle markers indicate when the CoM velocity was zero, representing the transition from the unweighting to the propulsive phase. The asterisks (*) and horizontal bars at the top of each graph indicate the time points showing significant correlations with the jump height. The asterisks and bars represent positive correlations, whereas the bars alone represent negative correlations.

Biology Open • Supplementary information

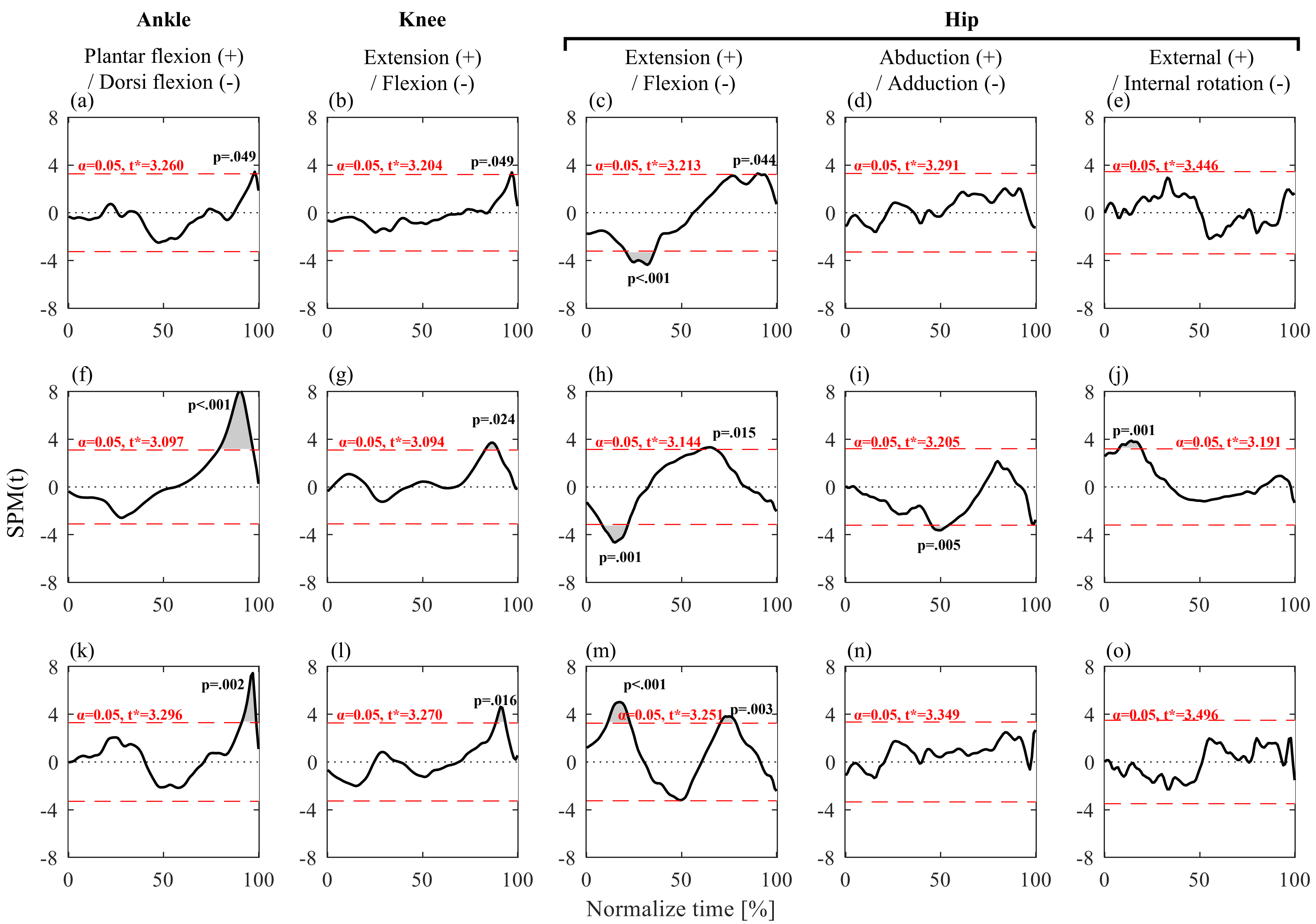

Fig. S7. The statistical results for joint angular velocities (a-e), torques (f-j), powers (k-o) of the ankle (a, f, k), knee (b, g, l), and hip (c-e, h-j, m-o) joints during the single-leg CMJ on the inferior leg side.

Biology Open • Supplementary information

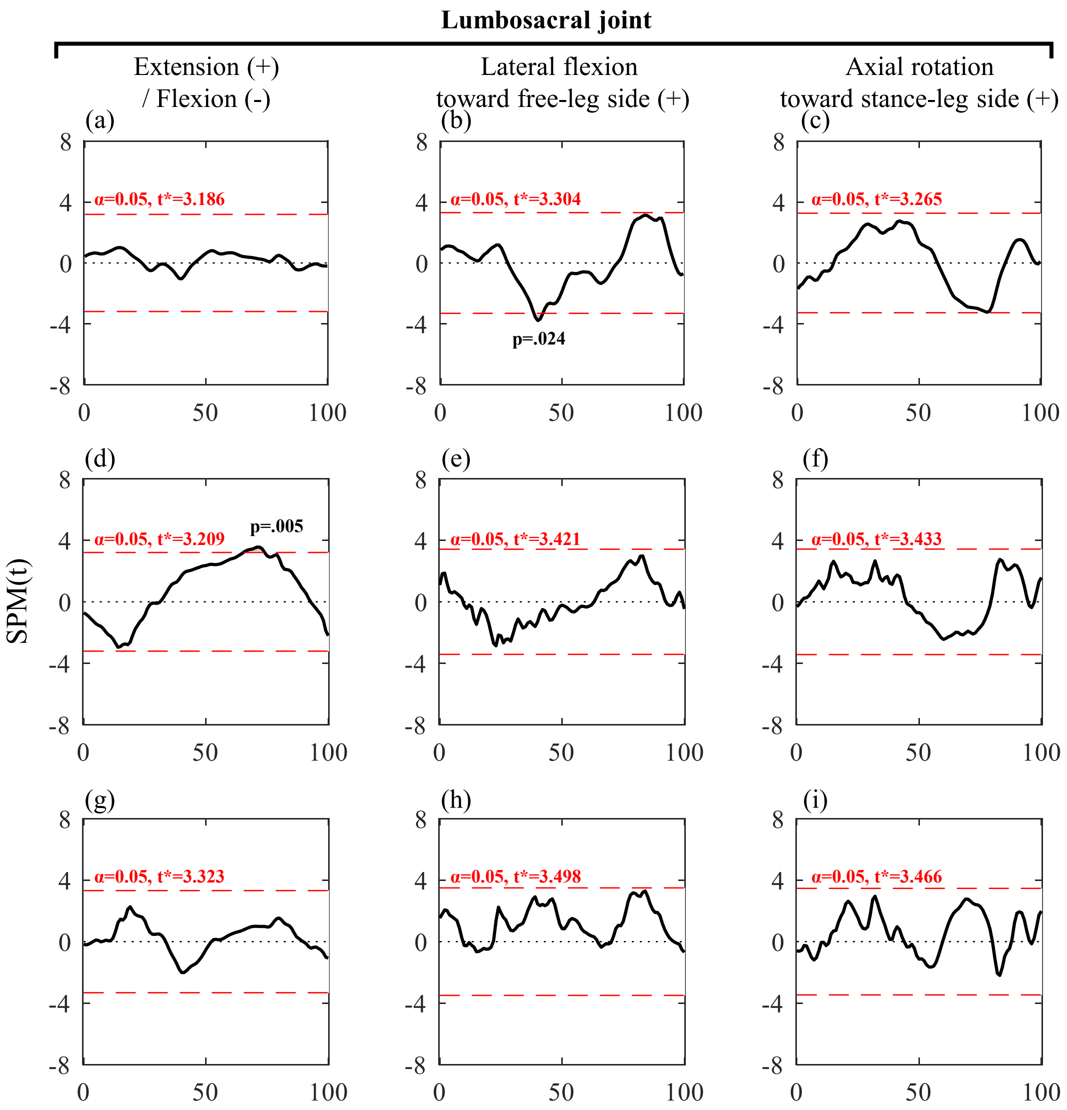

Fig. S8. The statistical results for joint angular velocities (a-c), torques (d-f), powers (g-i) of the lumbosacral joints during the single-leg CMJ on the inferior leg side.

Biology Open • Supplementary information
